# Indirect Microcontact Printing to Create Functional Patterns of Physisorbed Antibodies

**DOI:** 10.3390/s18093163

**Published:** 2018-09-19

**Authors:** Augusto Juste-Dolz, Miquel Avella-Oliver, Rosa Puchades, Angel Maquieira

**Affiliations:** 1 Instituto Interuniversitario de Investigación de Reconocimiento Molecular y Desarrollo Tecnológico (IDM), Universitat Politècnica de València, Universitat de València, 46022 Valencia, Spain; aujusdol@etsia.upv.es (A.J.-D.); miavol@upvnet.upv.es (M.A.-O.); rpuchades@qim.upv.es (R.P.); 2 Departamento de Química, Universitat Politècnica de València, 46022 Valencia, Spain

**Keywords:** microcontact printing, physisorption, diffraction-based sensing, label-free, antibody, immunoglobulin E, allergy, soft lithography, denaturation, paratope

## Abstract

Microcontact printing (µCP) is a practical and versatile approach to create nanostructured patterns of biomolecular probes, but it involves conformational changes on the patterned bioreceptors that often lead to a loss on the biological activity of the resulting structures. Herein we introduce indirect µCP to create functional patterns of bioreceptors on solid substrates. This is a simple strategy that relies on physisorbing biomolecular probes of interest in the nanostructured gaps that result after patterning backfilling agents by standard µCP. This study presents the approach, assesses bovine serum albumin as backfilling agent for indirect µCP on different materials, reports the limitations of standard µCP on the functionality of patterned antibodies, and demonstrates the capabilities of indirect µCP to solve this issue. Bioreceptors were herein structured as diffractive gratings and used to measure biorecognition events in label-free conditions. Besides, as a preliminary approach towards sensing biomarkers, this work also reports the implementation of indirect µCP in an immunoassay to detect human immunoglobulin E.

## 1. Introduction

Creating nanostructures of bioreceptors (proteins, nucleic acids, cells, etc.) nowadays plays an important role in biosensing, and it constitutes a significant trend in advances in nanoscience and nanotechnology [1,2]. Classical approaches to fabricating nanostructures for bioanalysis rely on placing biomolecular probes on solid nanomaterials [3,4,5,6,7]. Alternatively, developing nanostructures constituted by the bioreceptors themselves patterned on unstructured substrates is an appealing option in terms of simplicity, multiplexing, label-free capabilities, and cost-effectiveness, among others [8,9,10,11,12].

**Figure 1:**
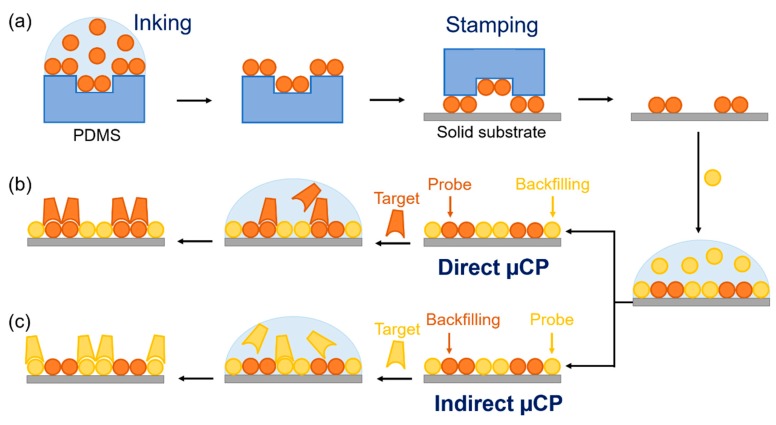
Schematic illustration of the fabrication and biorecognition processes, including (**a**) inking and stamping, (**b**) standard microcontact printing (µCP), and (**c**) indirect µCP. Note that in standard µCP, probes are patterned by stamping, whereas in indirect µCP, backfilling agents are stamped first and then the probes are physisorbed on the gaps just by incubation.

Contact techniques (pin printing, flow printing, dip pen nanolithography, etc.) enable well-defined and homogenous patterning, and they are typically employed to produce these structures of bioactive molecules [13,14]. Among these techniques, micro-contact printing (µCP) holds an important scientific relevance from its development. It relies on “inking” the probes on the surface of a nanostructured elastomeric stamp (typically made of polydimethylsiloxane) and then precisely transferring them to a solid material just by contact (Figure 1a). This versatile technique allows for the creation of large-scale patterns of probes of different natures (small molecules, proteins, nucleic acids, etc.) in standard biochemical lab settings [15,16,17], and with a resolution of up to about 50 nm [18]. Thanks to all these strengths, µCP holds a great popularity [19,20,21,22]. Nanometric patterns of bioreceptors fabricated by µCP have found many applications, such as microarraying and neuronal cells guidance among others [12,15,23]. Besides, these nanostructures also allow us to exploit nanoscopic light-matter phenomena to transduce biorecognition events, as is the case for diffraction-based sensing (DBS).

DBS relies on patterning biological probes as diffractive gratings on solid substrates, and then quantifying biorecognition events by the means of changes in their diffracted pattern. These changes come from variations in the mass that conforms the gratings (generated by probe–target interactions), and they can be easily quantified by measuring the intensity of a diffracted order [24,25,26,27]. This strategy allows for label-free sensing and real-time biorecognition assays of different natures [24,25], using simple assay setups [27], and in a multiplexed and high-throughput fashion [26]. Besides, µCP has shown to be a powerful technique to create functional nanostructures of bioreceptors for DBS [25,26,27,28,29].

µCP also enables direct patterning of proteins by physisorption. However, while this fact presents advantages in terms of simplicity, the conformational changes experimented by the patterned proteins along the physisorption process may lead to an important loss on their activity [30]. The conformation of physisorbed proteins on solid surfaces is governed by a complex interplay of different forces (Van der Waals, electrostatic, hydrogen bonding, conformational entropy, etc.) [14,31], which makes it extremely difficult to predict the functionality of a particular protein that is adsorbed on a given surface. This complexity increases even more when the proteins are patterned by standard µCP, since it involves two sequential adsorption processes, and the transfer to the final surface takes place in a rather dry state. This phenomenon was already observed in the early stages of µCP [32], and it is still an issue nowadays as it reduces the range of applications for this approach. An interesting alternative found in the state-of-the-art relies on mediating the patterning of proteins by self-assembled monolayers of reactive ligands structured by µCP [20,33,34]. This ligand-based strategy can provide solutions in terms of activity of the patterned proteins and introduces appealing options towards more stable immobilization chemistries, whereas on the other hand, it also limits the characteristic simplicity of µCP by introducing additional reagents and experimental stages.

The present study addresses this issue and focuses on creating functional structures of physisorbed antibodies by µCP. Given their central role in biosensing, antibodies were herein used as both, probes and targets. Also, since the functionality of their binding sites may be specially susceptible to the conformational changes involved in the structural rearrangements during the adsorption processes, these bioreceptors are highly suitable for the scope of this work. In fact, the results presented below highlight a substantial functionality difference between the paratopes and the epitopes of antibodies patterned by standard µCP. In this study, we explore this phenomenon on different materials using two model immunochemical systems and assessing by DBS and AFM the molecular-scale events taking place at the interface that holds the biomolecular pattern. From these results, this work proposes indirect µCP as a novel alternative to create functional structures of capture antibodies. This strategy keeps the characteristic simplicity of µCP, relies on physisorption, and provides a practical solution for those cases in which standard µCP leads to patterns of biomolecular probes with a reduced functionality. Herein we present indirect µCP and demonstrate it in a model immunochemical system, as well as in a label-free immunoassay to detect human immunoglobulin E.

## 2. Materials and Methods

### 2.1. Materials

Sodium phosphate buffer (PBS, 8 mM Na_2_HPO_4_, 2 mM KH_2_PO_4_, 137 mM NaCl, 2.7 mM KCl, pH 7.4), PBS-T (PBS with polysorbate 20 0.05% *v/v*), and carbonate buffer (50 mM sodium carbonate, pH 9.6) were prepared with purified water (Milli-Q, Millipore Iberica, Darmstadt, Germany) and filtered through 0.2 μm polyethersulfone membranes (Merck, Darmstadt, Germany). Polydimethylsiloxane (PDMS) Sylgard 184 was from Dow Corning (Wiesbaden, Germany). Bovine serum albumin (BSA), polysorbate 20 (Tween 20), polyclonal rabbit anti-BSA antibodies, polyclonal goat anti-rabbit antibodies (anti-RIgGs), and allyltrimethoxysilane were supplied by Sigma-Aldrich (Madrid, Spain). Rabbit antibodies (RIgGs) were purified from rabbit antiserum by affinity chromatography. Human immunoglobulins E (IgE) were from NIBSC (South Mimms, UK), and anti-IgE antibodies from Dr. Fooke (Neuss, Germany). Glass slides were purchased from Labbox (Mataró, Spain), polystyrene slides from Evergreen (Ted Pella Inc., Redding, CA, USA), and polymethyl methacrylate and polyester substrates were kindly supplied by Plexi (Valencia, Spain). Polycarbonate substrates were easily obtained from regular compact disks (MediaRange, MPO Iberica, Madrid, Spain) as described elsewhere [35]. The silicon grooved nanostructure (555.5 nm period, 140 nm groove depth, duty cycle 50%) used as a master for µCP was from LightSmyth (Eugene, OR, USA).

### 2.2. Patterning

Periodic nanostructures of biomolecular probes (proteins and antibodies) were fabricated on flat solid substrates by µCP. For that, PDMS (elastomer:curing agent, 10:1 *w*/*w*) was poured onto the nanogrooved side of the silicon master, degassed in a vacuum chamber for about 5 min, polymerized overnight at 60 °C, peeled off from the master, cut in 5 × 5 mm squared pieces, washed three times by sonication in ethanol (30% in water, 5 min), and dried under a stream of air. Then, as schematized in Figure 1, probe solutions in PBS (40 µL, 200 µg mL^−1^) were incubated on the structured side of the PDMS stamps during 160 min, the stamps were then rinsed with deionized water, dried by air stream, and stamped on the substrate. Finally, the chips containing the stamped structures were rinsed with water and dried as before.

**Figure A1:**
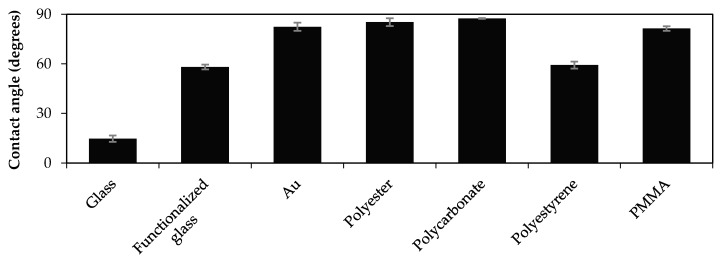
Contact angles of the different substrate materials studied in this work. Measured in water (2 µL droplets).

Prior to stamping, the substrate materials were washed three times by sonication in ethanol (30% in water, 5 min), and dried under a stream of air. Gold surfaces were created by sputtering (EM SCD005, Leica Microsystems, Madrid, Spain) a 50 nm thick layer of metal on polycarbonate chips. Functionalized glass was obtained by immersing slides in allyltrimethoxysilane (2% *v*/*v* in toluene) for 2 h under orbital agitation, rinsing the slides with toluene, drying them by air, and curing at 110 °C for 30 min. This silanization was monitored by contact angle measurements (Figure A1).

Two patterning strategies were performed in this work: standard (Figure 1b) and indirect (Figure 1c) µCP. The first one consists of the conventional state-of-the-art µCP process and it is based on an initial patterning of the probes as described in the paragraph above, and then incubating a backfilling agent to minimize non-specific binding during subsequent biorecognition assays. Conversely, indirect µCP relies on patterning first, backfilling (blocking) agent by standard µCP, and then incubating the bioreceptors on the patterned area (40 µg mL^−1^ in carbonate buffer, 2 h, 37 °C) in order to immobilize them by physisorption on the remaining gaps of the structure. In both cases, the resulting patterns were rinsed with deionized water and dried under a stream of air. A specific incubation of a backfilling agent was omitted in standard µCP, since previous reports showed that similar performances can be obtained in this case by just including polysorbate 20 in the incubation solution during the biorecognition steps [27].

### 2.3. Biorecognition Assays

To perform the biorecognition assays, 70–100 µL of sample containing target antibodies (0–100 µg·mL^−1^) in PBS-T were incubated on the bioreceptor nanostructures over 30 min at room temperature. Then, the chips were rinsed with PBS-T and deionized water, and they were dried under a stream of air. Custom circular incubation masks made of adhesive polymeric film were used to incubate the samples on glass. Three replicates for each condition were assayed and measured, and then used to calculate averaged and standard deviation values. Noise was estimated as the standard deviation from the measurement of 10 blank structures and employed to infer signal-to-noise ratios (SNR). Limits of detection were calculated as the concentrations associated with SNR = 3 from the linear interpolation in the experimental dose-response curves.

### 2.4. DBS Measurements

In essence, DBS responds to nanoscopic contrasts between the mass, constituting both parts of the diffractive structure (ridges and gaps in this case). This technique was herein employed to quantify biorecognition events, as well as to assess the characteristics of the developed nanometric patterns along the fabrication process. All the measurements were performed in air at endpoint conditions. Bioreceptor structures on transparent substrates (glass, functionalized glass, polystyrene, polycarbonate, polymethyl methacrylate, and polyester) were assessed by transmission, and by reflection on non-transparent materials (gold).

**Figure 2:**
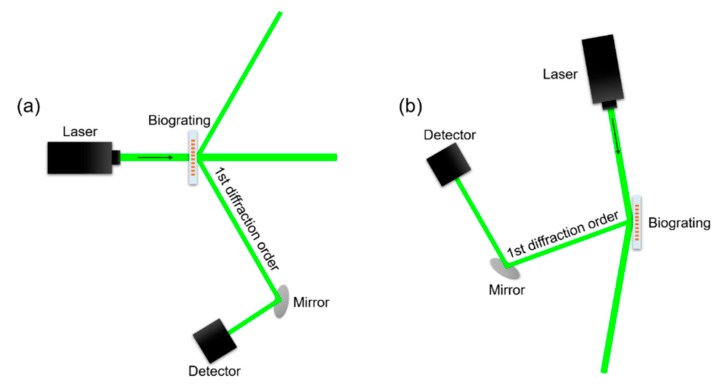
Schematic illustration of the experimental setups employed in this study to perform DBS measurements by (**a**) transmission and (**b**) reflection.

Transmission DBS measurements were performed using a simple optomechanical setup, as illustrated in Figure 2a. Basically, the chips containing protein structures were set to be orthogonally irradiated by a collimated and attenuated (95%) 532 nm laser source (100 mW, MGL-III-532, CNI, Changchun, China). This configuration generates a diffracted order whose intensity was measured using a monochromatic CMOS camera (1 ms of exposure time, Edmund eo-1312 m, York, UK). For reflection measurements, the setup was arranged to irradiate the surface at 80 °C and to collect the intensity of the diffracted order reflected from the surface, as illustrated in Figure 2b.

### 2.5. AFM Characterization

The topography of the developed bioreceptor structures was analyzed by atomic force microscopy (AFM), using tapping mode in air in a Multimode 8 microscope (Bruker) with RFESPA probes (MPP-21120-10 Bruker). AFM images were analyzed using Nanoscope software, and a first order polynomial flatten was applied in all the cases. To calculate the averaged cross section profiles, the area of interest was selected and the height of every data row along the longitudinal direction of the rides was averaged and plotted.

## 3. Results and Discussion

### 3.1. Substrate Materials

The nature of the substrate material is a key parameter for the performance of the physisorbed bioreceptors patterned by µCP. Regarding topography, suitable substrates must be flat enough to ensure maximal and homogeneous contact during the stamping stage. More importantly within the scope of this work, the chemical composition of the solid surface plays a crucial role on the conformational rearrangements undergone by proteins to become physisorbed, and therefore this parameter strongly affects their resulting functionality.

To explore this issue, this section evaluates (by DBS) the experimental dose-response curves of protein patterns fabricated by standard µCP on materials of different natures. For that, we first used a model system based on BSA as a probe and anti-BSA antibodies as targets. This is a reference immunoassay in the field of biosensing, herein selected to extend the scope of this assessment and to set up BSA patterning for its subsequent use as backfilling agent in this study (Section 3.2).

BSA patterns were fabricated on materials presenting different compositions and hydrophilicity (Figure A1), and a range of anti-BSA concentrations were incubated on them. Glass is a widely used substrate, and its functionalization (silanization) introduces interesting options to modulate surface properties [36]. Polystyrene is a well-known material for biosensing, and polycarbonate is interesting due to its good immobilization features and its compatibility with lab-on-a-disk biosensors [35]. Polymethyl methacrylate and polyester were selected as polymers for broadening the scope of this comparison. Regarding gold, it presents well-known probe immobilization features, and it is involved in many other label-free biosensing techniques (SPR, SERS, QCM, etc.).

**Figure 3:**
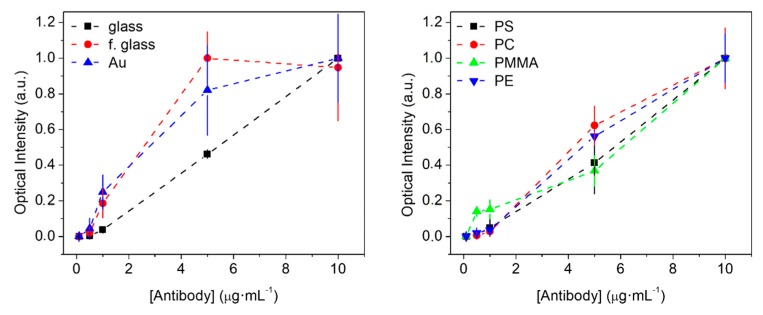
Dose-response curves for bovine serum albumin (BSA) probes and anti-BSA targets on different materials. Optical intensities in the vertical axes are normalized to the maximum intensity observed.

As observed in Figure 3, rather similar responses were obtained with all the different materials, and antibody concentrations of at least 0.5 µg mL^−1^ were detected in all the cases. More importantly, all dose-response curves display good correlations between the diffracted intensity and the target concentration. Although many processes may be involved in these responses (such as different physisorption densities, desorption processes, or non-specific binding, among others), this trend indicates that the conformations of the patterned protein are suitable to be recognized by the target IgG.

**Figure 4:**
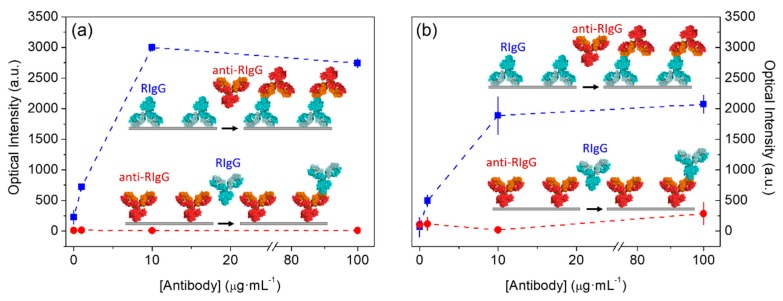
Dose-response curves of the immunochemical system based on rabbit antibodies (RIgG) and anti-rabbit antibodies (anti-RIgG) on (**a**) glass and (**b**) polycarbonate surfaces. In both cases, upper curves correspond to patterned RIgGs and anti-RIgG targets, and lower curves display the response of patterned anti-RIgGs and RIgG targets.

Beyond using BSA as probes, we extended this study to antibodies, since they play a central role in biosensing and their functionality may be highly dependent on the conformation of their physisorbed state. For that, we first patterned rabbit antibodies (RIgG) on glass, to be recognized by anti-rabbit antibodies (anti-RIgG). As observed in Figure 4a (upper curve), the response displayed by this system also indicates a suitable conformation of the patterned antibodies to be recognized by anti-RIgGs in solution. However, when these anti-RIgGs were patterned as probes, they were not able to bind their target RIgG anymore, and the corresponding dose-response curve displayed a negligible signal-concentration correlation (Figure 4a, lower curve). This observation indicates that the resulting conformation of these antibodies after the patterning process on glass strongly disrupts the functionality of their binding sites.

Then, the same experiment was conducted on polycarbonate. Since this polymer presents different compositional and hydrophilicity characteristics compared to glass (Figure A1), the patterned probes may lead to distinct conformations in their physisorbed state, which may change their functionality. However, equivalent results to glass were obtained with polycarbonate. A good correlation between concentration and diffracted signals was only obtained for patterned RIgGs on the substrate (Figure 4b, upper curve), but the dose-response for patterned anti-RIgG presents a rather flat trend (Figure 4b, lower curve).

Therefore, the results suggest that the functionality of these IgGs is much more sensitive to the conformational changes undergone during µCP if the patterned antibodies are the ones that recognize targets than if they are the ones being recognized (anti-RIgGs and RIgGs in this system, respectively). In other words, these conformational changes make paratopes (IgG regions that recognizes the antigen) become inactive when patterned, whereas the patterned epitopes (regions recognized by the paratopes) keep their ability to be recognized. Another potential explanation for these observations is that both paratopes and epitopes become strongly denatured when patterned, so paratopes become completely inactive, whereas some conformational epitopes remain unaltered and some lineal epitopes results accessible and can be recognized by certain paratopes within the polyclonal distribution of IgGs. In any case, it is interesting to highlight that this phenomenon may also potentially hinder the functionality of other biosensing configurations beyond this particular system, which would be interesting to explore in future investigations involving different biorecognition assays and patterning techniques.

### 3.2. Indirect µCP

As highlighted from the results in the section above, the paratopes of the antibodies patterned by standard µCP can undergo a dramatic decrease in their binding functionality. This problem is hard to predict and it constitutes an important limitation of µCP that may arise when developing new biosensing systems based on the nanostructured patterns of bioreceptors. Along these lines, in this section we introduce indirect µCP as a patterning alternative to address this issue.

As illustrated in Figure 1, indirect µCP relies on first patterning a backfilling agent by standard µCP. The denaturation of this agent does not affect on the performance of the assay, since it is merely used for backfilling. More importantly, this initial step leads to structured gaps on the surface, in which the desired bioreceptors can be immobilized by physisorption just by incubating them in solution. As a result, the biomolecular probes of interest can be immobilized in a structured fashion without undergoing all the conformational changes involved in µCP (only the ones in standard physisorption), thus experimenting milder processes and therefore presenting a lower potential to denature their binding sites and decrease their functionality. Furthermore, this strategy provides bioreceptor networks that already comprises an effective backfilling, which is very important to minimize potential artifacts coming from non-specific binding in the prospective use of these structures to analyze targets in complex matrixes [37].

**Figure A2:**
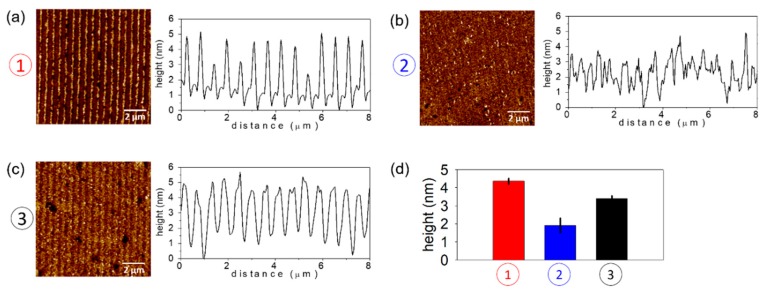
AFM topographic characterization along the different stages of indirect µCP: (**a**) Initial BSA patterning, (**b**) probe anti-IgG patterning, and (**c**) incubation of target IgG (100 µg mL^−1^). Left figures are the experimental AFM images and right graphs are their corresponding averaged cross section profiles. (**d**) Averaged height differences (ridges–grooves) obtained from the AFM scans above. The numbers in circles indicate the state of the patterns at each stage, according to Figure 5a.

To assess indirect µCP, we patterned BSA as a backfilling agent on glass, and then immobilized anti-RIgG as binding probes to subsequently recognize the RIgG targets in solution. As demonstrated in the section above, BSA presents great capabilities to be patterned by µCP on different materials and measured by DBS, and this fact, together with its inexpensiveness, makes this protein a highly suitable backfilling agent for this indirect printing. The experimental topographic characterization after this BSA patterning shows that the resulting structure is constituted by parallel, periodic, and straight ridges (Figure A2a), whose definition is affected by some smears and heterogeneities. These ridges present an averaged height difference (ridges-grooves) of about 4 nm (Figure A2d). Considering the prolate ellipsoid shape of BSA (14 nm for the polar axis and 4 nm for the equatorial axis), this height suggests that the protein tends to form a monolayer with its equatorial axis oriented orthogonally to the surface.

**Figure 5:**
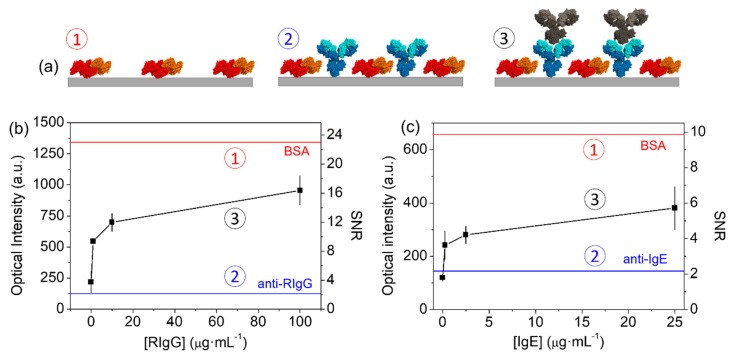
Experimental results for indirect µCP. (**a**) Schematic illustration of the state of the nanostructures ① after patterning the backfilling agent, ② after incubating the probe, and ③ after binding target antibodies. (**b**,**c**) Dose-response curves obtained from antibody structures patterned by indirect µCP for (**b**) anti-RIgG probes and RIgG targets, and (**c**) anti-IgE probes and human IgE targets. Upper and lower horizontal lines show the signal levels before and after incubating probe antibodies, respectively. The numbering of the curves indicates the state of the patterns at each stage, according to Figure 5a.

Figure 5b shows that the incubation of probes led to a dramatic decrease on the diffraction intensity, compared to the one of the initial BSA gratings. This fact points out that this incubation diminishes the difference in the mass that constitutes both parts of the grooved structure, which matches with the proper physisorption of the probes in the gaps, by a lot. This observation is also supported by the topographic characterization of the structure in this stage, where the averaged height difference decreases from 4.4 to 2 nm (Figure A2b,d). The not-null-diffracted signal obtained after incubating the probe must be generated by a greater mass in the anti-RIgG ridges, which agrees with the higher molecular weight of antibodies versus BSA (150 and 66.5 kDa, respectively). In fact, the AFM images also showed that the BSA ridges were narrower than the resulting gaps (Figure A2a), whereas the higher ridges after incubating the probe were actually the wider ones (Figure A2b).

Then, we studied the biosensing capabilities of these backfilled antibody structures by incubating different concentrations of target RIgGs in solution. Figure 5c shows the rising trend in the diffracted intensity obtained for increasing concentrations of target. This observation indicates that the conformation of the patterned paratopes preserve enough functionality to recognize the target epitopes. This proper recognition is also observed in the topographic measurements, which reaches an averaged height increase of 1.5 nm in the wider ridges (Figure A2c,d). A limit of detection of about 0.4 µg·mL^−1^ is inferred from the experimental dose-response curve in Figure 5c for this particular immunochemical system. Although higher diffracted intensities were registered when patterning RIgGs by standard µCP (Figure 4b, upper curve), it must be took into account that the indirect approach involves stronger backfilling conditions. Therefore, it results in much smaller contrasts between the mass conforming the probe and the mass in the backfilled ridges, which generates lower signals along the dose-response curve. However, what is important to highlight from these findings is that the presented indirect patterning strategy keeps the advantages of µCP and allows for the easy creation of functional structures of physisorbed antibodies that preserve the functionality of their paratopes, even for IgGs with a negligible functionality when patterned by standard µCP (lower curves in Figure 4b,c).

### 3.3. IgEs Immunosensing

To provide insights into the potential of indirect µCP for label-free biosensing, we implemented this approach in an immunochemical system to quantify human IgEs by DBS. IgEs are an isotype of immunoglobulins found in mammals, whose concentration in human blood serum is low at normal levels. This kind of immunoglobulins can be used as biomarkers for the diagnosis of allergies, parasitosis, immunoregulatory diseases, infections, and inflammatory disorders [38,39,40,41].

We developed diffractive nanometric patterns of anti-IgE antibodies on glass by indirect µCP, with BSA backfilling as before. The results presented in Figure 5c illustrate that the incubation of these antibodies on the BSA patterns also leads to an important decrease of the diffracted signal, which indicates the proper immobilization of the antibodies on the structure gaps, as discussed in the section above (Figure 5a,b). Furthermore, these results show that the diffracted signal increases together with the target IgE concentration, which indicates that the paratopes of the resulting anti-IgE nanostructures are also functional for this immunochemical system. From the results shown at Figure 5c, an experimental limit of detection of 0.2 µg·mL^−1^ of IgEs was inferred. Given the rather low SNR values obtained in this curve, this assay would be more suitable for qualitative analysis. For the particular application of this system in the diagnosis of allergies, this sensitivity enables for the detection of IgE concentrations in the range of ultra high levels of allergy, according to the radioallergosorbent test scores.

## 4. Conclusions

This work introduces indirect µCP as a strategy to create functional nanostructures of antibodies immobilized by physisorption. The experimental evidences herein presented demonstrate that, in some instances (typically hard to predict), these functional antibody gratings cannot be obtained by standard µCP. The results also suggest that the paratopes of immobilized antibodies are more prone to loose their activity after µCP patterning, with respect to their epitopes. Along these lines, BSA is a suitable protein as a backfilling agent for indirect µCP on a wide range of materials. When used to mediate the patterning of IgGs, antibody nanostructures with functional paratopes can be successfully fabricated by indirect µCP. This approach has proved its capabilities to create functional patterns of antibodies in a model system based on IgGs as probes and analytes, as well as in an immunoassay to detect human IgEs in label-free conditions by DBS. From these results, this study aims to introduce indirect µCP as a practical alternative for those cases in which standard µCP leads to patterns of non-functional biomolecular probes.
